# The Selective Removal of Bisphenol A Using a Magnetic Adsorbent Fused with Bisphenol A-Binding Peptides

**DOI:** 10.3390/ma17071651

**Published:** 2024-04-03

**Authors:** Yue Xu, Yujie Wu, Bharat Bhargawa, Soon Ho Hong, Ik-Keun Yoo

**Affiliations:** 1 School of Chemical Engineering, University of Ulsan, Ulsan 44610, Republic of Korea; 2 School of Chemistry and Chemical Engineering, Shanghai University of Engineering Science, Shanghai 201620, China

**Keywords:** bisphenol A, magnetic bead, adsorption, peptide

## Abstract

The potential of bisphenol A (BPA)-binding peptides fused to magnetic beads is demonstrated as novel adsorbents that are reusable and highly selective for BPA removal from aqueous environments, in which various interfering substances coexist. Magnetic beads harboring peptides (peptide beads) showed a higher BPA removal capacity (8.6 mg/g) than that of bare beads without peptides (2.0 mg/g). The BPA adsorption capacity of peptide beads increased with the number of peptides fused onto the beads, where monomeric, dimeric, or trimeric repeats of a BPA-binding peptide were fused to magnetic beads. The BPA-adsorbing beads were regenerated using a methanol–acetic acid mixture, and after six regeneration cycles, the adsorption capacity remained above 87% of its initial capacity. The selective removal of BPA was confirmed in the presence of BPA analogs with high structural similarity (bisphenol F and bisphenol S) or in synthetic wastewater. The present work is a pioneering study that investigates the selective affinity of peptides to remove specific organics with high selectivity from complex environmental matrices.

## 1. Introduction

Bisphenol A (BPA) has been widely used in the production of polycarbonate and epoxy resins [1,2]. It is a representative endocrine disruptor, which is principally released into the environment through its manufacturing processes, wastewater treatment effluents, and landfill leachates (e.g., hydrolysis of polycarbonate, recycled paper, etc.). Consequently, BPA can affect humans through food and drinking water intake, so there is a need to remove BPA from wastewater before it is discharged into the environment. These characteristics of BPA have increased public concern regarding its potential environmental risks. For example, the acute toxicity of BPA is in the range of 1–10 mg/L for several aquatic organisms [3]. Additionally, exposure to BPA at low levels of <1 μg/m^3^ can adversely affect human health. Human BPA exposure affects the male reproductive function and disrupts the thyroid function, which is associated with metabolic diseases, including diabetes and obesity, hypertension, and cardiovascular diseases [1]. To date, various methods have been investigated to handle BPA pollution, including advanced oxidation, adsorption, biodegradation, membrane separation, and photodegradation [4,5,6,7,8,9,10]. However, the efficiency of BPA removal in chemical oxidation and conventional bioprocesses varies with operating conditions [11,12]. Biodegradation and sludge adsorption have been reported as the two main mechanisms contributing to BPA removal in bioprocesses, with sludge adsorption achieving over 50% BPA removal efficiency [12]. However, large volumes of BPA-containing sludge pose challenges for treatment facilities, thereby demanding alternatives to fully address BPA contamination.

Adsorption is one of the preferred methods for treating water pollution because it effectively removes pollutants while offering the advantages of low operating costs and less harmful byproduct generation [13]. Various adsorbents have been studied for the adsorptive removal of BPA [4,14]. However, most adsorbents remove BPA in a nonselective manner and are therefore hindered by pollutants that coexist with BPA, thereby reducing their net BPA adsorption capacity and increasing the adsorbent’s cost. There are also additional challenges in the disposal of adsorbent residues containing the adsorbed BPA.

These challenges can be addressed by developing easily separable adsorbents with specific affinity for targeted contaminants. Peptides, which are oligomers of amino acids, are known to have selective affinity for specific targets. Recently, the use of peptides in environmental remediation has received great attention [15]. Although several peptide sequences have demonstrated affinity for specific targets [16], only a few peptide molecules can recognize low-molecular-weight organic compounds such as BPA. For instance, a cysteine-flanked heptapeptide, Cys–Lys–Ser–Leu–Glu–Asn–Ser–Tyr–Cys (CKSLENSYC), was screened through biopanning using a combinatorial constrained peptide library on the surface of M13 phage, displaying an affinity for BPA [17]. Magnetic particles can be rapidly collected under an external magnetic field, making them good candidates for reusable adsorbents [18,19,20,21]. By binding functional ligands with affinity to target molecules to magnetic particles, target compounds can be selectively removed from complex environmental matrices. There have been investigations on the adsorptive removal of BPA using magnetic particles. Magnetic graphene oxide impregnated with polymers (polystyrene, chitosan, and polyaniline) was applied for the adsorptive removal of BPA. This adsorbent showed a relatively high BPA adsorption capacity of about 36.27–86.22 mg/g, but its selective removal for BPA was not validated [20]. Magnetic graphene oxide-based molecularly imprinted polymer was studied for the selective removal of BPA from aqueous solutions, and the selectivity removal ratios of BPA over BPA structural analogs such as 2,4-DCP and phenol were 1.77 and 2.40, respectively, but the degree of structural similarity between BPA and 2,4-DCP or phenol was not sufficient enough to fully validate the selective removal performance of adsorbent [21].

In this study, we investigated the adsorptive removal of BPA using magnetic beads fused with selective BPA-binding peptides. The performance of the peptide-based adsorbent was evaluated at different pH levels, BPA concentrations, and peptide concentrations on the adsorbent surface. The selective removal of BPA by these peptide-based adsorbents was ensured in the presence of BPA structural analogs (e.g., bisphenol S (BPS) and bisphenol F (BPF)) or synthetic wastewater. To further increase the BPA removal capacity, adsorbents fused with multimeric peptides, such as dimeric and trimeric peptide repeats, were tested. Finally, the adsorption isotherm correlations were quantified to characterize the developed adsorbents.

## 2. Materials and Methods

### 2.1. Materials

Two forms of BPA-binding peptide (NH2–CKSLENSYC–COOH), a cyclic constrained form through a disulfide bridge between the flanking cysteine residue and a linear form, were custom-synthesized at Bio-FD&C (Lugen Sci., Bucheon, Republic of Korea). Linear forms of dimeric and trimeric peptides (NH2–KSLENSYKSLENSY–COOH and NH2–KSLENSYKSLENSYKSLENSY–COOH) were also synthesized. Peptides (100 mg/L) were dissolved in 100 mM MES buffer and stored at 4 °C before use. AccuBead^TM^ COOH magnetic beads with functional carboxyl groups (silica-coated magnetic beads, average diameter 1.56 µm, zeta potential −39.8 mV, 2.74 × 10^8^ COOH/bead) were purchased from Bioneer Inc. (Daejeon, Republic of Korea). The 4-morpholineethanesulfonic acid (MES), a buffer with a pKa value of 6.15, N-(3-dimethyl aminopropyl)-N′-ethylcarbodiimide hydrochloride (EDC), N-hydroxy-succinimide (NHS), bovine serum albumin (BSA, A7906), anhydrous acetonitrile (ACN), BPA, BPF, and BPS were obtained from Sigma-Aldrich (St. Louis, MO, USA). A BPA stock solution (1000 mg/L) was prepared in methanol and was diluted with water when used in the experiments. 

### 2.2. Construction of Peptide Bead

AccuBeadTM COOH magnetic beads were washed twice with a 25 mM MES buffer. Then, the BPA-binding peptide was covalently linked to the bead surface via carbodiimide-mediated amide bond formation (EDC/NHS activation) between the N-terminus of peptides and the carboxylic acid groups on the bead surface according to the Thermo Fisher Scientific protocol [22], with slight modifications. In detail, 3 mg of bare beads were incubated with 50 μL of EDC and 50 μL of NHS (each at 50 g/L in cold 25 mM MES) under slow tilt rotation for 30 min. Then, the carbodiimide-activated beads were collected using a magnet and washed with 25 mM MES. Next, 100 μL of the peptide (100 mg/L) was incubated with carbodiimide beads for 1 h. The peptide-linked bead was magnetically collected, and the peptide concentration in the supernatant was measured to determine the amount of peptide bound to the beads. For comparison, carbodiimide beads without peptide binding and beads coated with albumin protein were prepared using the same protocol. 

### 2.3. Bisphenol A Adsorption

After 500 μL of BPA (20 mg/L) was incubated with 0.3 mg of beads under slow tilt rotation for 1 h, the beads were collected through magnetic separation, and the BPA concentration in the supernatant was measured to determine the amount of BPA adsorbed on the beads. We investigated the change in BPA adsorption as a function of pH (2–10) and the change in BPA adsorption on beads prepared at different peptide doses (50–1000 mg/L). In bead reusability experiments, after adsorption, the beads were treated with a 500 μL methanol–acetic acid mixture (8:2, *v*/*v*) for 30 min and then washed with the 25 mM MES buffer. The amount of desorbed BPA was measured from the BPA concentration in the methanol–acetic acid solution, and these restored beads were consecutively reused for the subsequent adsorption rounds. The selective BPA-binding ability of peptide beads was evaluated using BPA analogs, such as BPS and BPF. The first set of adsorption experiments involved separate incubations of 0.3 mg of peptide beads with 500 μL of 15 mg/L BPA, BPS, or BPF solutions. The second set involved the incubation of peptide beads with a mixed solution composed of BPA, BPS, and BPF at equal concentrations (5 mg/L each, resulting in a total concentration of 15 mg/L). Finally, synthetic wastewater [23] containing BPA, BPS, and BPF was used to determine the BPA selectivity of peptides within a complex environmental matrix.

### 2.4. Analysis

Peptide concentrations were determined using high-performance liquid chromatography (HPLC, UV 220 nm, Agilent 1200, Santa Clara, CA, USA) with a C18 column (250 mm × 4.6 mm, 5 microns, Kromasil 100-5-C18, Kromasil, Göteborg, Sweden). A gradient elution of water containing acetonitrile and 0.1% (*v*/*v*) trifluoroacetic acid was used, ranging from 0% to 70% (*v*/*v*) acetonitrile at a flow rate of 1 mL/min. BPA concentrations were determined using another C18 column (Phenomenex 250 mm × 4.6 mm, Torrance, CA, USA) and a mobile phase of acetonitrile–water at a ratio of 57:43 (*v*/*v*) at a flow rate of 1 mL/min, monitored at 280 nm. The amount of BPA adsorbed per unit mass of adsorbent (*q_t_*) was calculated using the mass balance equation from the difference between the initial and final solute concentrations in the solution before and after adsorption, i.e., (1) *q_t_* = (*C*_0_ − *C_t_*)*V*/*W*, where *C*_0_ and *C_t_* are the initial and final BPA concentration in the solution (mg/L), respectively; *V* is the volume of BPA solution (L); and *W* is the adsorbent dosage (g). All analyses were performed in triplicate, and the obtained values were averaged.

## 3. Results

### 3.1. Preparation and Characterization of Peptide Beads

Peptide beads were constructed by linking the amine group of the peptide to the carboxylic group on the bead surface via an EDC/NHS reaction. NH2–CKSLENSYC–COOH, a previously screened BPA-binding peptide [24], was custom-synthesized in two conformations of the same sequence: a cyclic-constrained form (C-peptide) with a disulfide bridge between cysteine residues and a linear form (L-peptide) without a disulfide bridge. This differentiation aimed to examine the effect of the spatial conformation of peptides on their BPA-binding performance. Constraining a peptide via a disulfide bridge results in a structure with higher conformational stability than that of L-peptide, thereby improving affinity and selectivity for the target [25]. The amount of peptide bound to the beads was determined by measuring the peptide concentration in the supernatant after the EDC/NHS reaction between the peptide and beads. For instance, incubating 3 mg of EDC/NHS-activated beads with 100 μL of peptide (100 mg/L) resulted in slightly higher peptide binding in the C-peptide beads (1.73 × 10^8^ peptides/bead) than in the L-peptide beads (1.56 × 10^8^ peptides/bead). The manufacturer of the magnetic beads (AccuBead^TM^ COOH Magnetic Bead, Bioneer Inc., Daejeon, Republic of Korea) reported the number of carboxyl groups on the bead surface as 2.74 × 10^8^ –COOH/bead. Based on this value, the peptide linking efficiency to the bead was found to be at 56.9% (L-peptide) and 63.1% (C-peptide).

**Figure 1:**
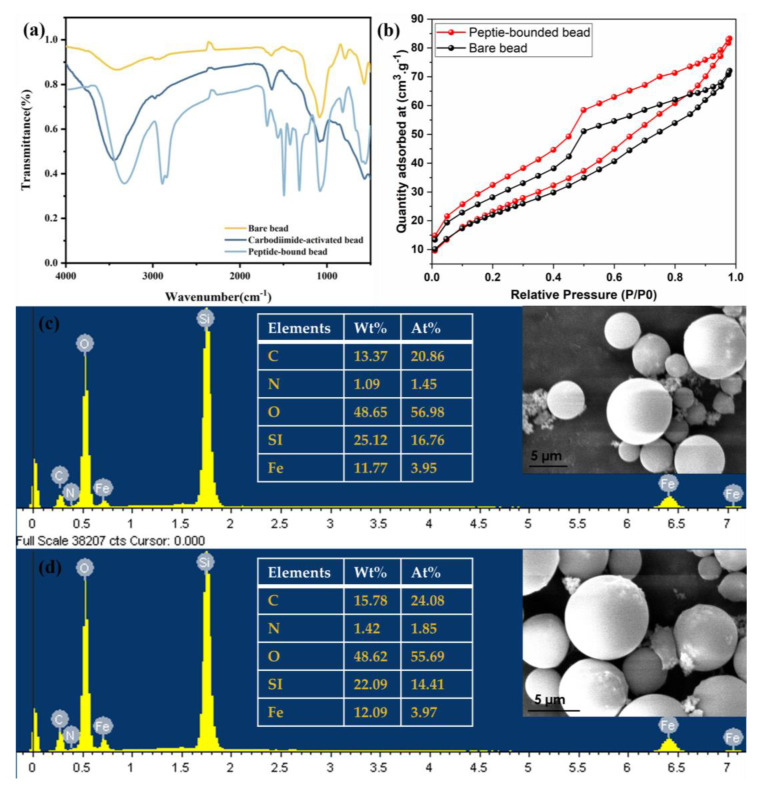
Fourier transform infrared (FT-IR) spectra of bare beads, carbodiimide-activated beads, and peptide beads (**a**); BET N_2_ adsorption-desorption analysis (**b**); SEM analysis of morphological differences in beads before and after Peptide adsorption (**c**,**d**).

Figure 1a displays a comparative analysis of Fourier transform infrared (FT–IR) spectra of bare, carbodiimide-activated, and peptide beads. The strong IR band observed at 580 cm^−1^ is a characteristic of Fe–O vibrations associated with the magnetic core, while the band centered at 1030 cm^−1^ corresponds to Si–O–Si or Si–O–Fe stretching vibrations of the silica shell. The strong IR bands located at 1553 and 1633 cm^−1^ are characteristic of amide bonds in peptide beads [26]. The strong C=O band observed at 1732 cm^−1^ clearly distinguishes bare beads from peptide beads, representing the C=O stretching vibrations of carboxylic acid. After peptide binding, a shift is observed, which is associated with covalent bonding between the peptide and the carboxyl group of the bead. The peaks centered at 1658 and 1546 cm^−1^ demonstrate coupling between N–H in-plane bending vibrations and C–N stretching vibrations of the peptide backbone, respectively, aligning well with previous reports [25,27,28]. The peaks located at 3305.8 and 1458.6 cm^−1^ are attributed to the stretching and in-plane bending vibrations of O–H in carboxylic groups. Moreover, characteristic absorption bands for –OH (–NH), –C=O, –CH_2_–/–CH_3_, and C–O ester/ether are located at 3458, 1732, 2974, and 1161–1259 cm^−1^, respectively. BET analysis was performed to evaluate the changes in bead surface area and pore volume before and after peptide adsorption. The adsorption–desorption results confirmed that the adsorbent follows a type IV adsorption isotherm with a characteristic hysteresis loop, as shown in Figure 1b, and the pores of the beads are in the mesoporous range. Compared to the bare beads, the peptide-bound beads exhibited an increase in surface area from 65.345 to 71.970 m^2^/g and an increase in pore volume from 0.101 to 0.118 cm^3^/g. This increase in surface area and pore volume is likely due to the attachment of peptide molecules to the bead surface. In the SEM-EDS analysis of the beads shown in Figure 1c,d, the bare bead surface was relatively smooth, but after peptide binding, the surface became rough. The changes upon peptide binding were also confirmed by EDS elemental analysis, where the presence of carbon and nitrogen increased from 20.86% to 24.08% and 1.45% to 1.85% atomic weight, respectively.

### 3.2. Bisphenol A Adsorption on Peptide Beads

**Figure 2:**
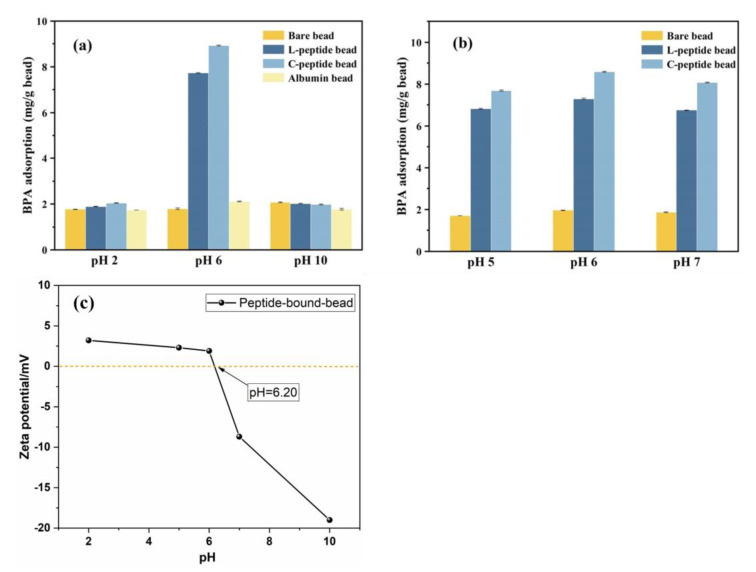
Comparison of BPA adsorption among bare beads, L-peptide beads, C-peptide beads, and albumin-coated beads at different pHs (**a**,**b**). Zeta potential of C-peptide bead at various pH values (**c**).

BPA adsorption is related to pH, as the pH of the solution influences the degree of BPA ionization, surface charge, and the extent of the dissociation of functional groups on the adsorbent [29]. The degree of the dissociation of BPA and peptides in the solution can be affected by the pH owing to the hydroxyl groups in BPA and amine and carboxyl groups in peptide beads. Figure 2a,b show a comparison of the BPA adsorption on bare beads, albumin-coated beads, L-peptide beads, and C-peptide beads at various pH values. No substantial differences in adsorption efficiency were observed between the four bead types at pH 2 and 10. However, the L-peptide and C-peptide beads showed considerably higher BPA adsorption capacities at pH 6 than the other two beads. The adsorption capacities of L-peptide bead, C-peptide bead, bare bead, and albumin-coated bead were 7.74, 8.93, 1.80, and 2.11 mg/g, respectively. Since C-peptide showed better adsorption capacity than others, C-peptide beads were used in subsequent experiments. 

The surface charge of a protein is positively charged when the pH is below the isoelectric point (pI) of the protein and negatively charged at a pH above the pI value [30]. The pI value of the peptide used in this study was calculated to be 6.1, rendering it positively charged at pH 2 and negatively charged at pH 10. Furthermore, BPA has a pKa of 9.9, from which the percentage of molecular BPA (nonionized form) can be calculated. BPA usually exists in molecular form below pH 7 and becomes negatively ionized at other pH levels [31,32]. Thus, electrostatic repulsion is expected to exist between the negatively charged peptide and BPA at pH 10, while minimal electrostatic repulsion is expected between the positively charged peptide and neutral BPA at pH 2. Consequently, the lower BPA adsorption efficiencies at both pH levels indicate an insignificant influence of electrostatic interactions on BPA adsorption. Similarly, the BPA adsorption efficiency on polymer adsorbent is improved when BPA exists in its molecular form [33,34]. Herein, to find the optimal pH condition, experiments were further conducted in the pH range of 5–7 (Figure 2b), thereby achieving slightly higher BPA adsorption capacity using the peptide bead adsorbent at pH 6. Around pH 6 (close to the pI value of the peptide), the electrostatic forces between the peptide and BPA weaken, allowing other interactions, such as π–π dispersion and hydrogen bonding, to facilitate BPA adsorption on peptide beads [13]. Hydrogen bonding can occur between the hydroxyl groups of BPA and the hydrophilic groups (carboxyl and hydroxyl) of the peptide, while π–π dispersions may exist between the benzene ring of BPA and the phenolic rings of the peptide. We also performed zeta potential analysis of the peptide beads, as shown in Figure 2c. The pH at which the peptide beads maintained zero net charge (pH-PZC) was calculated to be 6.2. This indicates that the adsorbent is negatively charged above pH 6.2 and positively charged below pH 6.2. The high adsorption near pH 6–7 is likely due to the stable electrostatic attraction between the positively charged BPA and negatively charged peptide beads. Additionally, previous studies on the ability of peptides to recognize specific targets reported that several factors, such as the sequence and local conformation of peptides and their coordination, are jointly involved in the binding of the peptides to specific targets [35,36,37,38]. Therefore, more independent studies are needed to address the interaction mechanism between BPA and peptides. 

**Figure 3:**
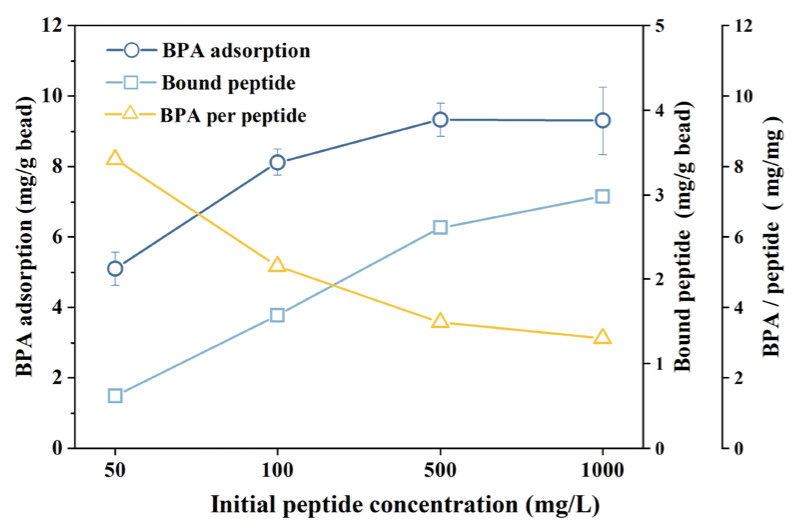
The amount of peptide bound to a bead in peptide beads constructed with different peptide doses; the amount of BPA adsorbed per unit bead mass; and the amount of BPA adsorbed per unit peptide mole.

Considering the correlation between the number of peptides on the bead surface and BPA adsorption capacity, the EDC/NHS reaction was conducted with different peptide doses (initial peptide concentration ranging from 50 to 1000 mg/L) to construct peptide beads. Figure 3 shows the moles of peptide bound to beads per unit bead mass in peptide beads, the moles of BPA adsorbed per unit bead mass, and finally, the moles of BPA adsorbed per unit peptide mole calculated using these two values. Upon increasing the peptide dose to 500 mg/L, the amount of peptide bound to the beads increases, but it becomes evident that peptide binding to the bead saturates within the 500 and 1000 mg peptide/L range. The total amount of BPA adsorbed to a bead saturates at ~500 mg/L, and eventually, the amount of BPA adsorption per mole of peptide decreases as the peptide dose increases to 1000 mg/L. Consequently, BPA adsorption is related to the number of peptides acting as BPA-binding sites. The decrease in BPA adsorption efficiency per mole of the peptide can be attributed to steric hindrance associated with the increased peptide density on the bead surface.

**Figure 4:**
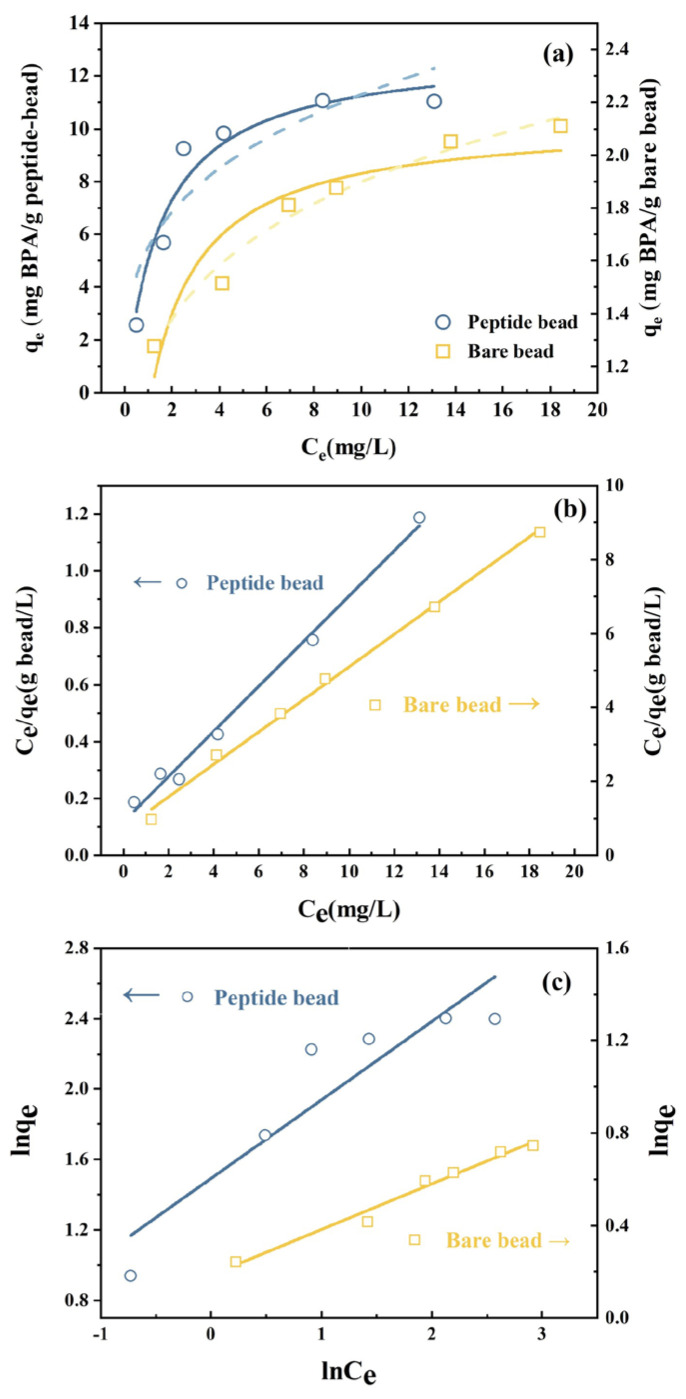
BPA Adsorption isotherms on peptide beads and bare beads: (**a**) nonlinear fitting using Langmuir (solid lines) and Freundlich isotherms (dash lines); (**b**) linear Langmuir isotherm; (**c**) linear Freundlich isotherm.

To quantitatively analyze BPA adsorption by peptide beads, adsorption isotherm experiments were conducted while varying the initial BPA concentrations from 2 to 20 mg/L. The L-shaped adsorption isotherm for peptide beads in Figure 4a shows that the Langmuir adsorption model (Equation (1)), which describes uniform adsorbate distribution (BPA) on the uniform adsorbent surface (peptide beads), fits better than the Freundlich model (Equation (2)), which describes multilayer adsorption on heterogeneous adsorbent surfaces. Conversely, in the case of bare beads, the isotherm data fit the Freundlich model better than the Langmuir model. To calculate the model parameters, we applied the linearized Langmuir model (Equation (3)), as depicted in Figure 4b, and the linearized Freundlich model (Equation (4)), as illustrated in Figure 4c.

(1) $$q_{e}=\frac{q_{max}K_{L}C_{e}}{1+K_{L}C_{e}},$$

(2) $$q_{e}=k_{f}C_{e}^{1/n},$$

(3) $$\frac{C_{e}}{q_{e}}=\frac{1}{q_{max}}C_{e}+\frac{1}{q_{max}K_{L}},$$

(4) $$lnq_{e}=lnk_{f}+\frac{1}{n}lnC_{e},$$

where *q_e_* (μmol/g of adsorbent) is the equilibrium BPA adsorption capacity of beads, *C_e_* (μmol/L) is the equilibrium BPA concentration in the solution after adsorption, *k_f_* is the Freundlich constant, and 1/*n* is the heterogeneity factor. The empirical parameters *q_max_* (μmol/g) and *K_L_* (L/μmol) denote the theoretical maximum adsorption capacity of the adsorbent and the affinity constant, respectively [39].

Linear regression analysis showed that the Langmuir model (R^2^ = 0.930) provided a better fit for the peptide beads than the Freundlich model (R^2^ = 0.767). In contrast, the Freundlich model (R^2^ = 0.974) was a better fit for bare beads than the Langmuir model (R^2^ = 0.845). This is because BPA adsorption onto peptide beads occurs in a monolayer form through selective peptide binding, whereas BPA adsorption onto bare beads occurs through a nonselective, randomized, and multilayer binding approach. The Langmuir parameters for the peptide beads were determined as a *q_max_* of 13.0 mg/g bead and *K_L_* of 0.64 L/mg, while the Freundlich parameters for the bare beads were determined as a *k_f_* of 1.20 mg/g bead and *n* of 5.04.

### 3.3. Reusability of Peptide Bead

**Figure 5:**
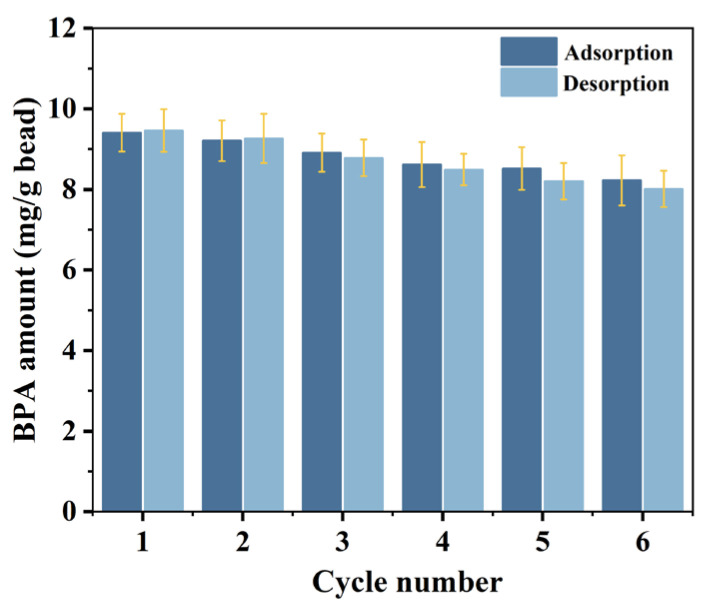
BPA adsorption and desorption over six adsorption–desorption cycles.

The reusability of the adsorbent is an important property in terms of cost reduction. Considering previous studies [40,41], a methanol solution was first tested as a desorbent for BPA from the beads, but the BPA desorption efficiency was unsatisfactory. In contrast, Bayramoglu et al. reported a high BPA desorption ratio (up to 98%) from molecularly imprinted polymers [42], prompting us to explore a methanol–acetic acid mixture for elution after various trials. During six adsorption–desorption cycles, we monitored the regeneration of the peptide beads used for BPA adsorption. Figure 5 illustrates that an accurate mass balance between the adsorbed and desorbed BPA was not achieved owing to an incomplete desorption process. The amount of adsorbed and desorbed BPA gradually decreased during the successive use of the beads. The adsorption capacity of the regenerated beads in the sixth cycle (8.22 mg/g) remained over 87% of the initial adsorption capacity compared to that in the first operation. Although much research is needed to find better eluents and conditions to minimize the loss of adsorption capacity, limited studies have reported the optimal BPA adsorption–desorption conditions.

### 3.4. Selective Bisphenol A Adsorption

**Figure 6:**
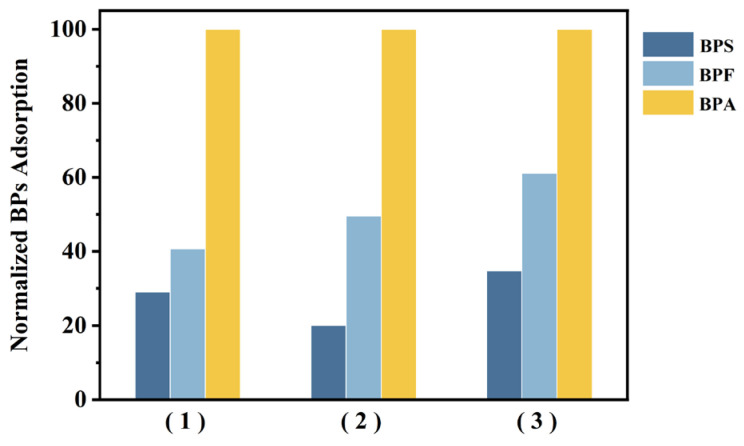
Comparison of BPA adsorption and adsorption of BPA analogs (BPF and BPS) under various conditions: (1) single-component solution; (2) mixture of BPA, BPF, and BPS; (3) synthetic wastewater containing BPA, BPF, and BPS.

BPA has several structural analogs, including bisphenol E (BPE), BPF, and BPS. A previous analysis of the removal characteristics of these bisphenols in a full-scale wastewater treatment plant [12] found that the average concentration of BPA (2.031 μg/L) in municipal untreated wastewater was much higher than that of BPA analogs (0.077 μg/L). The estrogenic potency of BPA is similar to or higher than BPA analogs, suggesting that focus should be primarily directed toward BPA compared to other BPA analogs. Adsorption studies were conducted with bisphenol analogs to evaluate whether the selectivity of peptide toward BPA, previously demonstrated on microbial surfaces [43], would be maintained in the context of a magnetic bead-based adsorbent. BPS and BPF were tested as interferences against BPA binding in single and mixed contaminant solutions. As shown in Figure 6, the constructed peptide beads exhibited the highest affinity for BPA, with markedly low adsorption of BPS and BPF in single-pollutant systems. Similar hydrogen bonds also could be formed between BPA analogs and the peptide owing to the structural resemblance (–OH) of BPS and BPF with BPA. Compared to BPS, BPF showed relatively higher binding capacity onto peptide beads in all experimental conditions, which is similar to a previous study involving a microorganism-based peptide adsorbent [43]. The selective affinity of a peptide for a particular target is determined by a complex interplay of factors related to the peptide’s amino acid composition, coordination chemistry, electrostatic properties, and pH [36]. The mechanism by which peptides interact with specific targets is not yet fully understood, and quantitative binding experiments and modeling can provide some clues as to how this is possible. Previous studies have assumed that the binding of BPA onto peptides occurs due to the interaction of BPA with the Ser 333 and Asn 336 residues of the peptide sequence. Hydrophobic interactions by polar aliphatic residues and H-bond interactions, especially by Asn, are important in the binding chemistry of the peptide with BPA [43]. Compared to the bare bead without peptide, relatively more BPA adsorption onto wild-type microorganisms (not possessing peptide) was observed, which is attributed to nonspecific binding to some surface peptides/proteins on the microorganism. To date, magnetic adsorbents for BPA removal have been developed using complexes of iron and other materials (activated carbon, cellulose, biochar, etc.), but only a few cases have been reported regarding the selective removal of trace organic pollutants [29,44,45,46]. 

To further evaluate the selectivity of peptide beads toward BPA, batch experiments were conducted by preparing a mixture of BPA, BPS, and BPF. Furthermore, peptide beads showed good selectivity for BPA in the mixture system. The adsorption capacities of peptide beads toward BPA, BPS, and BPF were 3.46, 0.76, and 1.50 mg/g, respectively, indicating that the BPA selectivity of peptide beads was maintained. Finally, to simulate real water environments, synthetic wastewater containing a mixture of the above three analogs was tested. Compared with the solutions above, the peptide bead retained its highest affinity for BPA; however, its BPA removal capacity slightly diminished when operating in the simulated wastewater. This reduction may be due to the presence of various organic and inorganic contaminants in the wastewater solution, which may bind to the beads and reduce the BPA adsorption capacity of the peptide beads. Nevertheless, the selectivity of BPA over BPS remained at 2.9, suggesting the versatility of the peptide beads as selective adsorbents in various environments. Molecularly imprinted polymers (MIPs) have commonly been investigated as selective BPA adsorbents. For example, photoresponsive MIPs based on mesoporous carriers, palygorskite-supported surface MIPs, and water-soluble MIPs showed excellent affinity and selectivity for BPA in aqueous solutions, but some MIPs required rather complicated synthesis processes [47,48,49].

### 3.5. Bisphenol A Adsorption Using Different Lengths of Peptide Repeats

**Figure 7:**
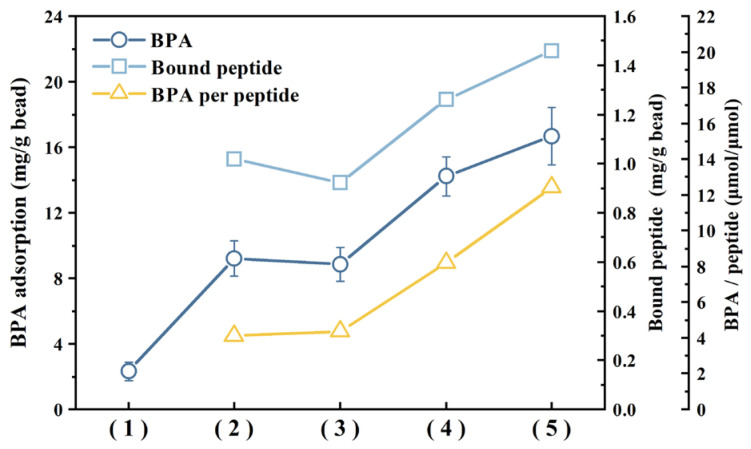
Comparison of BPA selective adsorption capacities using peptides of different lengths: (1) are bead; (2) C-peptide bead; (3) L-peptide bead; (4) dimeric L-peptide bead; (5) trimeric L-peptide bead.

In a previous study of BPA adsorption on recombinant cells, a dimeric display of the BPA-binding peptide on the cell surface was employed to improve BPA adsorption [50]. The dimeric strain exhibited a BPA adsorption rate of 230.4 µmol BPA/g DCW at a BPA concentration of 15 ppm, which was threefold higher than the monomeric strain. To see if the magnetic bead-based adsorbent could exhibit similar performance, we constructed beads linked to dimeric and trimeric repeats of the linear peptide to further enhance the BPA removal capacity of peptide beads. Although the C-peptide demonstrated higher binding to the beads, resulting in higher BPA removal efficiency than the L-peptide (Figure 2), for this experiment, the beads were constructed using the L-peptide. This is because the C-peptide could not be synthesized in the repeated cyclic forms. As shown in Figure 7, BPA adsorptions of bare, C-peptides, L-peptide, dimeric L-peptide, and trimeric L-peptide beads were 2.32, 9.22, 8.84, 14.24, 16.66 mg/g, respectively. As shown in Figure 7, the mass ratio of bound peptide to bead (mg/g) also increased with the increase in peptide length in the order of monomeric, dimeric, and trimeric L-peptide beads, but the number of bound peptides per bead decreased as the molecular weight of dimeric and trimeric peptides increased by a factor of two and three, respectively. Therefore, the ratio of adsorbed BPA to monomeric, dimeric, and trimeric peptide beads was determined as 4.34, 8.22, and 12.42 μmol BPA/μmol peptide, respectively. This observation means that the amount of BPA adsorbed on peptide beads is proportional to the number of monomeric peptides that have an affinity for BPA, eventually confirming that BPA adsorption is mainly caused by the binding affinity between BPA and BPA-binding peptide. In future studies, it is necessary to investigate methods for increasing the number of peptides on the bead surface to further improve the BPA removal capacity.

## 4. Conclusions

The selective affinity of peptides has been well studied, but their practical application has been limited to binding to metals and metal oxides, with little application to organics such as BPA. This work is a pioneering study in the application of peptides with high selective affinity for organics to the removal of specific organic pollutants. Molecularly imprinted polymers have also been studied for the selective removal of BPA and have the advantage of large adsorption capacity. However, their selective affinity for BPA in the presence of BPA analogs with high structural similarity (bisphenol F and bisphenol S) has not been fully validated. We created a reusable adsorbent with a peptide with a specific affinity for BPA linked to magnetic beads. The adsorption capacity (8.6 mg/g) of this peptide bead adsorbent occurred at pH 6 and fit well with the Langmuir isotherm model. This peptide adsorbent exhibited remarkable selectivity for BPA compared to its analogs. We also compared BPA adsorption for monomeric, dimeric, and trimeric repeats of the BPA-binding peptide and found that BPA adsorption was proportional to the number of each monomeric BPA-binding peptide. In a reusability study, the BPA adsorption capacity after six cycles retained more than 87% of the initial adsorption capacity. These validations demonstrated that the developed peptide-based magnetic adsorbent has actual potential for selective BPA removal from complex environmental matrices, especially in environments where excessive amounts of adsorbent are required to remove BPA coexisting with interfering pollutants.

## Data Availability

Data are contained within the article.
